# Evaluation of serum Nestin and HOTAIR rs12826786 C>T polymorphism as screening tools for breast cancer in Egyptian women

**DOI:** 10.5937/jomb0-25295

**Published:** 2021-01-26

**Authors:** Sarah A. Aglan, Mohamed Elsammak, Omar Elsammak, Eman A. El-Bakoury, Heba G. Elsheredy, Yasser S. Ahmed, Mohamed H. Sultan, Ahmed M. Awad

**Affiliations:** 1 Alexandria University, Medical Research Institute, Department of Chemical Pathology, Egypt; 2 Alexandria University, Faculty of Medicine, Egypt; 3 Alexandria University, Medical Research Institute, Department of Radio-diagnosis, Egypt; 4 Alexandria University, Medical Research Institute, Department of Cancer Management and Research, Alexandria, Egypt; 5 Alexandria University, Medical Research Institute, Department of Experimental and Clinical Surgery, Alexandria, Egypt

## Abstract

**Background:**

Nestin is a neural stem cell protein that plays an important role in cancer stem cells (CSC) development and proliferation. It has been identified as a marker for newly formed endothelial cells and was shown to be preferentially expressed in basal and myoepithelial cells of the mammary gland. *HOTAIR* is long intergenic non-coding (linRNA) associated with tumorigenesis through promotion of epithelial-mesenchymal transition (EMT) and stemness as well. *HOTAIR* gene contains a functioning single nucleotide polymorphic site rs12826786 C>T that has been associated with several cancer types.

**Methods:**

We evaluated serum Nestin and the *HOTAIR* rs12826786 C>T polymorphism in healthy Egyptian women and those with breast cancer as a possible screening tool to identify patients with breast cancer. Also, we tested the possible association of the two markers with each other and the aggressiveness of the disease.

**Results:**

Patients with breast cancer had a median (Min-Max) of serum Nestin 31.3 (6.7-167.3 pg/mL), while control subjects had a median (Min-Max) of serum Nestin 42.3 (25.7-315.95) pg/mL. The best cut-off value for serum Nestin to differentiate normal subjects and patients with breast cancer was 39.9 pg/mL. This cut-off value had a diagnostic sensitivity of 84.8% and specificity of 65.1%. There was a significant difference in the distribution of different alleles in patients with breast cancer than normal subjects (P=0.039 Exact Fisher test). The breast cancer patients group had 23.9% CC, 52.1% CT, and 23.9% TT genotypes, respectively, while the control group had 46.9% CC, 42.8% CT, and 10.2% TT, respectively.

**Conclusions:**

A significantly low serum Nestin below 39.9 pg/mL and a higher percentage of the T/T homozygous variant allele of *HOTAIR* rs12826786 C>T were found in Egyptian patients with breast cancer. We suggest that the reported cut-off value of serum Nestin and the presence of C/T polymorphism can be used to assess the risk of females for developing breast cancer and might be of potential benefit in screening the disease. Larger studies in different ethnic groups are needed to confirm our findings.

## Abstract (sr)

**Uvod:**

Nestin je protein neuronskih matičnih ćelija koji ima važnu ulogu u razvoju i proliferaciji matičnih ćelija raka (CSC). Identifikovan je kao marker za novoformirane endotelne ćelije, a pokazalo se da je preferencijalno izražen u bazalnim i mioepitelnim ćelijama mlečne žlezde. *HOTAIR* je dugo intergensko nekodiranje (linRNA) povezano sa genezom tumora kroz promociju epitelno-mezenhimske tranzicije (EMT) kao i karakteristikama matičnih ćelija. *HOTAIR* gen ima jedno funkcionalno nukleotidno polimorfno mesto rs12826786 C > T koje je povezano sa nekoliko tipova raka.

**Metode:**

Izvršena je evaluacija serumskog Nestina i HOTAIR rs12826786 C>T polimorfizma kod zdravih žena u Egiptu, kao i onih sa karcinomom dojke, kao mogućeg skrining alata za identifikaciju pacijenata sa karcinomom dojke. Takođe, testirali smo moguću povezanost između ova dva markera i agresivnosti bolesti.

**Rezultati:**

Pacijentkinje sa karcinomom dojke su imale srednju vrednost (Min-Maks) seruma Nestina 31,3 (6,7-167,3 pg/mL), dok su kontrolne ispitanice imale srednju vrednost (Min-Maks) seruma Nestina 42,3 (25,7-315,95 pg/mL). Najbolja granična vrednost Nestina u serumu koja je ukazivala na razliku između zdravih ispitanica i pacijentkinja sa karcinomom dojke bila je 39,9 pg/mL. Ova granična vrednost imala je dijagnostičku osetljivost od 84,8% i specifičnost od 65,1%. Bilo je značajne razlike u distribuciji različitih alela kod pacijentkinja sa karcinomom dojke nego kod zdravih ispitanica (P=0,039 Fišerov test tačne verovatnoće). Grupa bolesnica sa karcinomom dojke imala je 23,9% CC, 52,1% CT i 23,9% TT genotipa, dok je kontrolna grupa imala 46,9% CC, 42,8% CT i 10,2% TT respektivno.

**Zaključak:**

Kod egipatskih pacijentkinja koje su imale karcinom dojke otkriven je značajno nizak serumski Nestin, ispod 39,9 pg/mL, i veći procenat alela homozigotne varijante T/T HOTAIR rs12826786 C>T. Predlažemo da se prijavljena granična vrednost Nestina u serumu i prisustvo C/T polimorfizma koristi za procenu rizika kod žena za razvoj raka dojke, a može biti i od koristi u skriningu bolesti. Potrebne su obimnije studije u različitim etničkim grupama da bi se naši nalazi potvrdili.

## Introduction

Breast cancer remains the second most diagnosed cancer after lung cancer worldwide, with an incidence of 11.6% of all cancers. It accounts for 6.6% of all cancer-related deaths (5^th^ among all cancer types), according to GLOBOCAN 2018 [1]. Epidemiological studies show that the incidence of breast cancer is even increasing in developing countries [2]. Early diagnosis and detection, markedly improves survival in affected subjects. The new treatment modalities can achieve nearly 100% five years of survival [3]. Most societies advocate screening for breast cancer starting around the age of 40 [4]. The recommended screening modality for breast cancer is mammography (digital or film) [5]. Screening for breast cancer using mammography can detect breast cancer and save many lives; however, it has its downsides. First, the sensitivity for the detection of breast lesions is affected by breast tissue density, being less sensitive in cases where the breast tissue is denser [6]. Second, the sensitivity of mammography to breast lesions increases with the increase of the age of patients. In other words, mammography is not very sensitive at young age [7]. Furthermore, radiation exposure, although the dose of radiation is small but repeated, may pose high risk, questioning the reasoning of screening of females at young age using mammography [8]. Moreover, expensive radiology equipment is needed, and expert interpretation of x-ray films is required, adding more to the need of early safe markers to screen and evaluate the risk for developing the disease in younger female population.

Currently, CA15.3 is the widely used breast tumor marker; however, it is not recommended for screening purposes. It is only used for follow up and prognosis in already diagnosed patients [9]. Thus the presence of biochemical markers that can be checked by a single blood test seems to be an attractive idea for breast cancer screening and early detection.

Nestin is a neural stem cell protein that plays an important role in cancer stem cells (CSC) development and multiplication [10]. It has been identified as a marker for newly formed endothelial cells and was shown to be preferentially expressed in basal and myoepithelial cells of the mammary gland [11]. Cancer stem cells were identified in different types of cancers, including breast cancer [12]. They are capable of self-generation and proliferation. Cancer stem cells play a pivotal role in tumor differentiation, progression, tumor angiogenesis, and tumor cell migration and invasion [12].

Several studies suggested that Nestin expression is directly proportional to the activity and properties of cancer stem cells in different tumors with high expression, cor relating to poor prognosis [13].

A recent study based on Western patients has shown that Nestin is preferentially expressed in basallike breast carcinomas, predominantly expressed in triple-negative breast cancers, and suggested the use of Nestin as a marker for triple-negative breast cancer [14]. A recent meta-analysis concluded that Nestin positivity and expression was associated with shorter overall survival [13].

Furthermore, a recent study found a positive correlation between Nestin mRNA and Nestin protein expression in germline *BRCA1* related breast cancer, a basal-like phenotype, with reduced survival, and stem ness characteristics [14]. The marker expression was reported in endothelial cells of breast as well [15].

So far, Nestin has been evaluated in breast cancer patients only on a tissue level using an immunohistochemistry technique for diagnostic and prognostication purposes. Up to our knowledge so far, no study has evaluated serum Nestin in patients with different stages of breast cancer compared to healthy control women.

On the other hand, the role of genetics in the development of cancer has been widely acknowledged with several genes mutations, which are now well known predisposing factors for the development of different types of cancer (e.g., BRCA and breastovarian cancer) [16] [17]. Several genetic loci have also been found to correlate with the susceptibility to cancer [18] [19].

Long non-coding RNA sequences had been identified as RNA sequences that are longer than 200 bases [20]. Several of these long non-coding RNA (lnRNA) sequences participate in the control of different cellular processes. Disturbance in the regulation of any of these processes (e.g., cell cycle, proliferation, expression of different proteins) may increase the susceptibility to different types of cancers [21].

Hox transcript antisense intergenic RNA (*HOTAIR*) is a long intergenic non-coding RNA spanning more than 2 kb with 6 exons. Its gene is located on chromosome 12 between *HOXC11* and *HOXC12* genes [22]. The RNA is transcribed from the antisense DNA strand, and it exerts its function in trans. *HOTAIR* mediates methylation of H3K27 and demethylation of H3K4 through recruiting and binding to polycomb repressive complex 2 (PRC2) and Lysine-specific histone demethylase 1(LSD1) leading to dynamic changes in histones and subsequent target gene silencing [23].

Alves et al. [24] highlighted in their study the role of *HOTAIR* in epithelial-mesenchymal transition (EMT) and promotion of stemness in colon and breast cancer cell lines, they reported a loss of EMT in those cell lines upon ablation of expression of *HOTAIR* through siRNA. They also reported a higher level of *HOTAIR* expression, particularly in colon cancer stem cells (CSC) than other non-stem cell subpopulations [24], a similar finding that was also reported in oral carcinoma by Lu et al. [25].

Different studies illustrated the association between* HOTAIR* SNPs and the genetic susceptibility to various cancers such as ovarian, GIT, and breast cancer [26] [27] [28].

The effect of* HOTAIR* rs12826786 polymorphism on *HOTAIR* expression was examined by Guo et al. [27] study who found a higher expression of linRNAs *HOTAIR* in gastric cardia adenocarcinoma tissue carrying TT genotype of *HOTAIR* rs12826786 than in tissues carrying CC wild type.

The current study aimed to evaluate serum Nestin as well as *HOTAIR* rs12826786 C>T polymorphism in Egyptian women. The study also aimed at evaluating the possibility of using Nestin and *HOTAIR* gene rs12826786 polymorphism as possible screening tools to identify patients with breast cancer.

### Material and Methods

This case-control study included 46 patients with pathologically proven invasive breast cancer who presented to the Surgery, Cancer Management and Research and Radiodiagnosis Departments, Medical Research Institute, Alexandria University, Alexandria, Egypt, during February, March, and April 2019.

Subjects who had any history of ischemic heart disease, liver, or kidney disease and those who were taking antibiotics, on any kind of immunosuppressive medications or had any other form of primary cancer than the breast were excluded.

Besides, 49 age-matched normal healthy individuals were included in the study as a control group. All patients and controls were living in the same geographic area of Northern Egypt (Alexandria); all of them had the same lifestyle (none of them was a heavy smoker nor had excessive alcohol consumption).

All subjects gave informed consent before the study began, and the study was approved by the Medical Research Institute, Alexandria university Ethical committee, and according to the Helsinki declaration. All patients and controls had a full clinical examination, including history taking.

The control group comprised 49 women with negative screening mammography. Their age (Mean±SD) was 43±3.64 years. The patient group comprised 46 breast cancer women Aged (Mean±SD) 50.64±9.52 years.

Patients were subjected to preoperative evaluation, including history taking, clinical examination to detect the site of the tumour, and the presence of enlarged lymph nodes and metastatic workup.

Mammography was performed for enrolled females using a General Medical Italia (GMI) LAMB-DA series (15LAM11) analogue mammography unit with the following features: high-frequency x-ray generator, 20-35 kV-50kHz ripple with auto kV option, mAs range of 1-640, potter Bucky 24x30.

The routine mammographic views are obtained for each breast, mediolateral oblique (MLO) and craniocaudal (CC) views. Each mammographic study was interpreted by a radiologist with 15 years of experience.

Patients that showed suspicious mammographic findings i.e. masses, asymmetry or calcifications were recalled for ultrasound and then subjected to ultrasound guided core biopsy.

The diagnosis of invasive breast cancer was made by a core-needle biopsy of the breast tumour. The staging was defined by the eighth edition of the Cancer Staging Manual of the American Joint Committee on Cancer [29]. Breast core biopsies were histopathologically examined. Immuno histo chemical analysis (estrogen receptor (ER) progesterone receptor (PR)-Epidermal growth factor receptor 2 (HER2)) of the tumour tissue on core biopsies and lumpectomy/mastectomy specimens was done.

Formalin-fixed paraffin-embedded breast biopsies were examined microscopically, using standard lightmicroscopic evaluation of sections stained with Hematoxylin and Eosin in each case and classified according to WHO classification of breast tumor 4^th^ edition [30]. Grading was done according to the Nottingham modification of the Bloom-Richardson system [31]. Immunohistochemical examination of tissue sections was done for HER2 and scoring is according to American Society of Clinical Oncology/ College of American Pathologists Clinical Practice Guideline Update 2013 [32] and also done to evaluate PR/ER according to American Society of Clinical Oncology/College of American Pathologists 2010 guidelines [33]. Breast cancer cases were classified into the intrinsic molecular subtypes; Luminal A/B-HER2 over-expression and basal-like (triple-negative) [34]. Clinico-pathological data of patients was as follows; stage I: 5 (10.9%), stage II:13 (28.2%), stage III: 12 (26.1%), stage IV: 16 (34.8%). 16 (34.8%) cases were metastatic while 30 (65.2%) cases were non metastatic. All cases had invasive carcinoma of no special type (NST). 41 (89.1%) cases were grade 2, while grade 3 cases were 5 (10.9%). Tumor size >/= 5 cm were 33 (71.7%), while tumors < 5 cm were 13 (28.3%). Nodal positivity was present in 31 (67.4%) cases, while 15 (32.6%) cases had negative nodes involvement. 37 (80.4%) cases were Luminal A/B, 4 (8.7%) were HER2 over-expressing and 5 (10.9%) were triple negative. Cases with BI-RADs 3 were 1 case (2.2%), BI-RADs 4 were 24 (52.2%), BI-RADs 5 were 20 (43.5%), while those with BI-RADs 6 were 1 case (2.2%).

All blood samples were collected by venipunctures into serum and an EDTA containing vacutainer tube. Serum samples were obtained by centrifugation at room temperature for 10 minutes at 2000g. The serum aliquots were immediately frozen at -20 °C until use. Both groups had the following laboratory tests performed: ALT, triglyceride, cholesterol, and creatinine. Serum Nestin was carried out for both groups using commercially available ELISA human Nestin quantitation kit (Biomatik (Ontario, Canada, Cat no: PRS-02453hu).

### HOTAIR SNP analysis

### DNA Extraction

Genomic DNA was isolated from nucleated blood cells using DNA extraction from EDTA whole blood using Automated MagCore® Genomic DNA Whole Blood Kit, Cartridge Code 102 performed on MagCore®HF16 automatic extractor. DNA samples were kept at -20°C till analyzed.

### Genotyping using PCR-RFLP analysis

The *HOTAIR* gene variants were detected by PCR-RFLP analysis using FastDigest BglII (Thermofisher, USA) restriction analysis of a 226-bp polymerase chain reaction-amplified fragment in the *HOTAIR* gene cutting the variant at position 53961717 on Chromosome 12 after amplification of the target sequence spanning from 53961658 : 53961883 position. Primers were designed using Primer-BLAST, a free online NCBI tool, available at https://www.ncbi.nlm.nih.gov/tools/primerblast/inde x.cgi?LINK_LOC=BlastHome.

Amplification of target sequence was done using 50 to 80 ng DNA samples in a final volume of 25 µL containing 1×PCR buffer with 1.5 mmol/L MgCl_2_, 2 unit Taq DNA polymerase, 100 µmol/L dNTP, and 0.5 µmol/L of each primer (Forward: 5' - GTGAGAGAC-CTCCAAGAGCG-3' and Reverse: 5' - CTTGTCGAG-GCCCAGTTTCT-3'). PCR was performed using Labcycler SensoQuest GmbH (Göttin gen, Germany), and the profile of amplification consisted of an initial denaturation step at 94 °C for 3 minutes. This was followed by 40 cycles of Denatu ration at 94 °C for 30 seconds, Annealing at 59 °C for 30 seconds, and extension at 72 °C for 30 seconds. A final extension step at 72 °C for 3 min was carried out.

For restriction enzyme analysis, 10 µL of the amplified PCR product was enzymatically digested at 37 °C for 30 minutes with Thermo Scientific Fast-Digest BgIII restriction enzyme. The digestion mixture comprised 17 µL of nuclease-free water, 2 µL of fast digest buffer, and 1 µL of the BgIII restriction enzyme.

The restriction enzyme BgIII (Thermofisher, USA) was used to distinguish the wild allele (C) 226 bp from the variant allele (T) 166 and 60 bp. The gain of a BglII restriction site occurs in the variant allele. The wild genotype has a single band representing the entire 226-bp fragment, and the heterozygous genotype results in three fragments of 226, 166, and 60 bp, while the homozygous variant allele results in 2 fragments 166 and 60 bp. Finally, the products of the BgIII digestion were electrophoresed on 2% agarose gel, and the final products were visualized using ethidium bromide staining under UV light.

To ensure quality control, genotyping was performed blindly to case/control status, and negative samples (nuclease-free water) were amplified with each run to detect contamination.

### Statistical analysis

Statistical analysis of the data analysis was carried out using the Kruskal-Wallis test. Prevalence of alleles and genotype among cases and control subjects were counted and compared with Hardy-Weinberg predictions. Chi-square test (χ^2^, Fisher's exact test) was used to test the distribution of the different genotypes in the different groups. For correlation studies, the Pearson correlation test was used. A P-value of < 0.05 was considered statistically significant. For the choice of the optimal cut-off, receiver operating characteristic (ROC) curves were constructed, and the Youden index was calculated [35]. The Youden index is defined as follows: (sensitivity + specificity) -1. The best cut-off value has the highest Youden index. The commercial statistical software package used was SPSS 18.0 (SPSS, Inc, Chicago, IL, USA).

## Results

There was no significant difference between the studied group in serum, ALT, serum Creatinine, or general biochemistry results (P>0.05), data were not shown.

Serum Nestin followed a non-Gaussian distribution and was presented as median (range). Serum Nestin was significantly lower in patients with breast cancer compared to normal controls (P=0.032).

**Figure 1:**
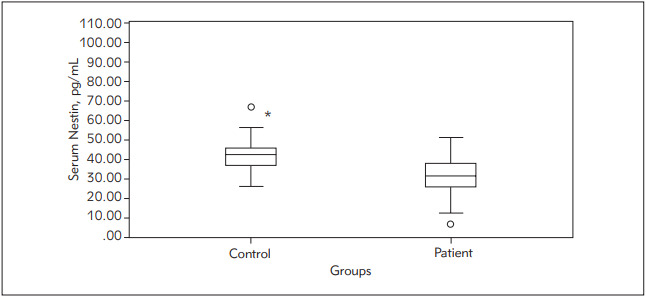
Serum Nestin (pg/mL) in the studied groups Boxplots illustrate serum Nestin (pg/mL) in patients with breast cancer and normal control subjects. The boxplots represent the interquartile range from the 25^th^ to the 75^th^ percentiles. The whiskers below and above the boxes represent the minimum and maximum values, respectively. The line across each box represents the median value. N = number of patients included in each group. * = significant difference between control subjects and breast cancer patients (P<0.05). Circle outlier (values larger than the upper quartile plus 1.5 times the interquartile range). One outlier in each group is not shown on this scale.

Figure 1 shows the distribution of serum Nestin patients with breast cancers and the control group. There was a significant difference between patients and controls. Serum Nestin ranged between 6.7 and 167.3 pg/mL with a median value of 31.3 in patients with breast cancer compared to a median of 42.3 and a minimum of 25.7 and a maximum of 315.95 pg/mL in normal control women.

Nestin was found to be in our study lower in birads 5 than 4 (almost all cases are birads 4 or 5 except 2 cases only were 3 and 6), with nestin value medians 28.9 and 33.7 pg/mL, respectively. Nestin was also lower in patients with large tumor size at presentation (>/= 5 cm) than small tumor size (tumor size taken at largest diameter); median 27, 31.6 pg/mL, respectively. Yet those findings did not reach statistical significance. There was no significant difference in serum Nestin between LN negative or LN positive patients as well. Similarly, serum Nestin did not show significant differences between patients with or without distant metastasis (data not shown). No association was observed between serum Nestin and molecular intrinsic subtypes, or tumor staging. Nestin was found to be slightly higher in patients with grade 3 (median of 37.5 pg/mL) than patients with grade 2 (median of 31.3 pg/mL). However, no statistical difference was detected, given the small number of patients with grade 3 (5 cases only).

**Figure 2:**
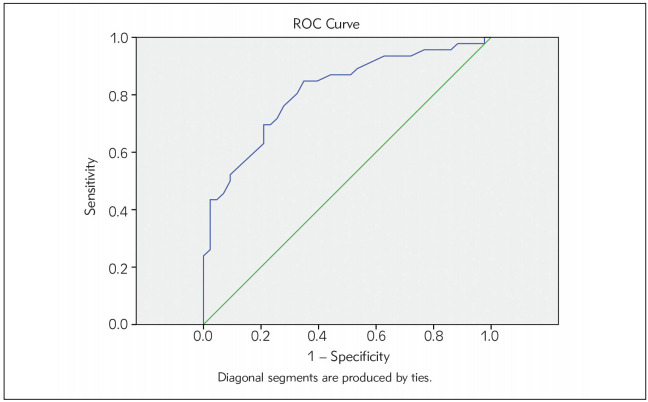
Receiver-operating characteristic (ROC) curves for serum Nestin ROC curves were constructed by plotting the sensitivity versus 1-specificity at different cut-off values for serum Nestin. The number of cases included in the study was 46 patients with breast cancer and 49 control subjects. The area under the curve (AUC) for serum Nestin was 0.812 (P-value = 0.000 and 95% confidence interval [CI] was (0.723–0.901).

The discriminating power of Nestin in discriminating normal from patients affected with breast cancer was tested by plotting a ROC curve. Figure 2 illustrates a ROC curve of serum Nestin. The area under the curve (AUC) for serum Nestin was 0.812 (P-value = 0.000 and 95% confidence interval [CI] was 0.723-0.901). Using the Youden index, the best cutoff value for serum Nestin to differentiate normal subjects and patients with breast cancer was 39.9 pg/mL. This cut-off value had a diagnostic sensitivity of 84.8% and specificity of 65.1%.

Healthy controls genotypes for HOTAIR gene SNP rs12826786 were distributed in accordance with Hardy-Weinberg equilibrium with x^2^ = 0.32 and P value = 0.57. The minor allele (T) frequency was 0.35.

**Figure 3:**
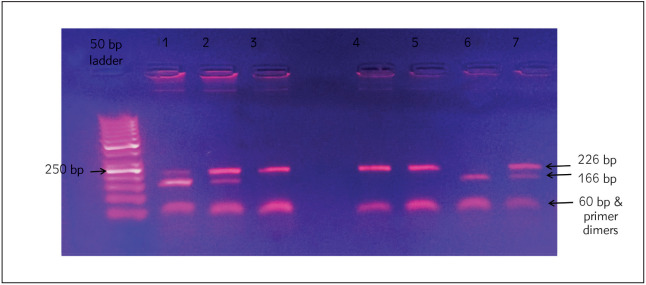
Different rs12826786 C>T HOTAIR genotypes 2% agarose gel electrophoresis showing the different genotypes of the HOTAIR rs12826786 C>T SNP. DNA samples were amplified using PCR and digested with FastDigest BgIII restriction enzyme. The presence of C to T polymorphism creates a FastDigest BgIII restriction site. 50 base pairs (Bp) ladder marker Lane 1, 2, 7 – Heterozygous genotype (C/T) Lanes 3, 4, 5 – Wild genotype (C/C) Lane 6 – Homozygous genotype (T/T)

Figure 3 shows the result of the enzymatic digestion of the *HOTAIR* gene. The wild *HOTAIR* allele has a 226bp while the heterozygous variant has 226, 166, and 60bp bands while the homozygous variant has two bands of 166 and 60 bp, respectively.

Table 1 shows the distribution of the *HOTAIR* gene genotypes in control subjects and patients with breast cancer. The breast cancer patients group had 23.9% CC, 52.1% CT, and 23.9% TT genotypes, while the control group had 46.9% CC, 42.8% CT, and 10.2% TT.

**Table 1:** Frequency of the *HOTAIR* genotypes in control subjects and patients with breast cancer The frequency of T/T allele in the breast cancer patients is higher than that observed in the normal controls. On the other hand, the C/C allele is at lower frequency than that in the normal controls. The result of the Chi-square testing of genotypes in the studied groups; Showed statistically significant difference among the groups (P = 0.039 Exact Fisher test).

*HOTAIR* Genotypes				
Group				Total
	C/C	C/T	T/T	
Control	23 (46.9%)	21 (42.8%)	5 (10.2%)	49
Patient	11 (23.9%)	24 (52.1%)	11 (23.9%)	46
Total	34 (35.8%)	45 (47.3%)	16 (16.8%)	95

There was a significant difference in the distribution of different alleles in patients with breast cancer than normal subjects (P = 0.039 Exact Fisher test). The T/T genotype was frequently observed in breast cancer patients, while C/C genotype had lower frequency in patients than in healthy controls. Different genotypes were not associated with tumor size, nodal status, staging, metastasis, molecular intrinsic subtypes, or grade of breast cancer. Serum Nestin level was not correlated with different genotypes of *H* *OTAIR* gene SNP rs12826786 (data not shown).

## Discussion

Nestin is considered a marker of stemness and has been linked to the proliferation of cancer stem cells and poor prognosis [10] [11] [13]. It is worth noting that Nestin was also thought to be abundantly expressed in regenerating areas and in endothelial cells (EC), and thus has been regarded as a marker of angiogenesis and proliferation associated with tumors. However, this dogma of Nestin being a marker of proliferation has been challenged by a recent study by Dusart et al. [15], where they demonstrated that the inhibition of expression of Nestin through siRNA led to a proliferation of endothelial cells. Also, they reported high expression of Nestin in EC across several organs among them was the breast, challenging the previous knowledge of the restricted expression and stemness of Nestin. Furthermore, they observed a lower expression of Nestin in EC of bladder urothelial carcinoma and lung adenocarcinoma tissue than in corresponding normal tissue, further demonstrating that Nestin is not associated specifically with EC of tumors [15].

In agreement of the aforementioned recent study observations by Dusart et al. [15], our study found a significantly lower serum Nestin values in breast cancer group compared to the control group, with a cut-off of 39.9 pg/mL representing the best value for discriminating patients with breast cancer from normal women, where, lower Nestin values were found to be associated with increased probability to cancer. This cut-off value had a sensitivity of 84.8% and specificity of 65.1%.

This finding is in agreement with Krüger et al. [36], who found significantly lower Nestin expression in breast cancer tissues of patients. They observed Nestin positivity in only 9-28% of studied hospital breast cancer patients. They hypothesized that increased Nestin expression is only limited to cases of basal intrinsic subtype that are highly undifferentiated and associated with Cytokeratin, P-cadherin, and EGFR staining [36].

Up to our knowledge, this study is the only study so far evaluated serum Nestin in a cohort of breast cancer patients.

The current study also found a significant association between the *HOTAIR* rs12826786 C>T polymorphism and breast cancer in Egyptian women. There was an increased percentage of the homozygous T/T allele in Egyptian women affected with breast cancer. Several studies reported the association between *HOTAIR* gene polymorphism and different diseases [22] [23] [24]. The finding of the current study is concordant with those of Bayram et al. [37], who reported the significant association between the *HOTAIR* rs12826786 C>T polymorphism and breast cancer in Turkish patients [37]. Similarly, a recent Iranian study reported similar findings in an Iranian cohort of breast cancer patients [38]. *HOTAIR* gene function includes the control of different processes in cell proliferation and cell cycle control. Given that *HOTAIR* rs12826786 C>T polymorphism leads to higher expression of *HOTAIR* [27] thus, the presence of the homozygous variant allele might be a predisposing or susceptibility factor for the development of breast cancer through enhancement of proliferation.

The current study did not report any association between the *HOTAIR* rs12826786 C>T polymorphism and serum Nestin.

In essence, the current study is the first of its kind to shed light on the pattern of serum Nestin in aspecific cohort of Egyptian patient affected with breast cancer. The study also specified a cut-off value of 39.9 pg/mL serum Nestin to discriminate patients affected with breast cancer from normal subjects. Furthermore, the study evaluated the *HOTAIR* rs12826786 C>T polymorphism in a sample of Egyptian patients.

The shortcomings of the current study involve a small number of patients studied in the breast cancer patient group. Future work will concentrate on reproducing the reported results in a bigger cohort of the breast cancer group to investigate serum Nestin and *HOTAIR* gene polymorphism in different subgroups of breast cancer patients according to their histopathological and molecular subtype.

## Conflict of interest statement

This work has been funded by a grant from Medical research institute research cancer research fund. The authors declare no conflict of interest.
